# Unique Features of *Aeromonas* Plasmid pAC3 and Expression of the Plasmid-Mediated Quinolone Resistance Genes

**DOI:** 10.1128/mSphere.00203-17

**Published:** 2017-05-24

**Authors:** Dae-Wi Kim, Cung Nawl Thawng, Sang Hee Lee, Chang-Jun Cha

**Affiliations:** a Department of Systems Biotechnology and Center for Antibiotic Resistome, Chung-Ang University, Anseong, Republic of Korea; b Department of Biological Sciences, National Leading Research Laboratory of Drug Resistance Proteomics, Myongji University, Yongin, Republic of Korea; Escola Paulista de Medicina, Universidade Federal de São Paulo

**Keywords:** *Aeromonas*, plasmid-mediated quinolone resistance, *aac*(*6'*)*-Ib-cr*, miniature inverted-repeat transposable element, *qnrS2*, regulator of sigma D

## Abstract

A highly fluoroquinolone-resistant isolate of *Aeromonas* species was isolated from a wastewater treatment plant and found to possess multiple resistance mechanisms, including mutations in *gyrA* and *parC*, efflux pumps, and plasmid-mediated quinolone resistance (PMQR) genes. Complete sequencing of the IncU-type plasmid, pAC3, present in the strain revealed a circular plasmid DNA 15,872 bp long containing two PMQR genes [*qnrS2* and *aac*(*6′*)*-Ib-cr*]. A mobile insertion cassette element containing the *qnrS2* gene and a typical miniature inverted-repeat transposable element (MITE) structure was identified in the plasmid. The present study revealed that this MITE sequence appears in other *Aeromonas* species plasmids and chromosomes. Plasmid pAC3 was introduced into *Escherichia coli*, and its PMQR genes were expressed, resulting in the acquisition of resistance. Proteome analysis of the recipient *E. coli* strain harboring the plasmid revealed that *aac*(*6′*)*-Ib-cr* expression was constitutive and that *qnrS2* expression was dependent upon fluoroquinolone stress through regulation by regulator of sigma D (Rsd). To the best of our knowledge, this is the first report to characterize a novel MITE sequence upstream of the PMQR gene within a mobile insertion cassette, as well as the regulation of *qnrS2* expression. Our results suggest that this mobile element may play an important role in *qnrS2* dissemination.

**IMPORTANCE** In the present study, plasmid pAC3 isolated from a highly fluoroquinolone-resistant isolate of *Aeromonas* species was sequenced and found to contain two fluoroquinolone resistance genes, *aac*(*6′*)-*Ib-cr* and *qnrS2*. Comparative analyses of plasmid pAC3 and other *Aeromonas* sp. IncU-type plasmids revealed a mobile insertion cassette element with a unique structure containing a *qnrS2* gene and a typical miniature inverted-repeat transposable element (MITE) structure. This study also revealed that this MITE sequence appears in other *Aeromonas* species plasmids and chromosomes. Our results also demonstrate that the fluoroquinolone-dependent expression of *qnrS2* is associated with *rsd* in *E. coli* DH5α harboring plasmid pAC3. Our findings suggest that the mobile element may play an important role in *qnrS2* dissemination and that *Aeromonas* species constitute an important reservoir of fluoroquinolone resistance determinants in the environment.

## Abstract (precis)

In the present study, plasmid pAC3 isolated from a highly fluoroquinolone-resistant isolate of *Aeromonas* species was sequenced and found to contain two fluoroquinolone resistance genes, *aac*(*6′*)-*Ib-cr* and *qnrS2*. Comparative analyses of plasmid pAC3 and other *Aeromonas* sp. IncU-type plasmids revealed a mobile insertion cassette element with a unique structure containing a *qnrS2* gene and a typical miniature inverted-repeat transposable element (MITE) structure. This study also revealed that this MITE sequence appears in other *Aeromonas* species plasmids and chromosomes. Our results also demonstrate that the fluoroquinolone-dependent expression of *qnrS2* is associated with *rsd* in *E. coli* DH5α harboring plasmid pAC3. Our findings suggest that the mobile element may play an important role in *qnrS2* dissemination and that *Aeromonas* species constitute an important reservoir of fluoroquinolone resistance determinants in the environment.

## INTRODUCTION

Fluoroquinolones are broad-spectrum antimicrobial agents that have been widely used to treat bacterial infections (1). Residual fluoroquinolones have been detected at various environmental sites (2), including wastewater treatment plants (WWTPs) (3), where antibiotics may act as a selective pressure that allows a potential genetic exchange of resistance genes (4, 5).

Bacterial resistance to fluoroquinolones has been demonstrated to be caused by (i) chromosomal mutations in the genes encoding target proteins such as DNA gyrase or topoisomerase IV (6), (ii) efflux pumps (7), (iii) Qnr family proteins (8), and (iv) inactivation by fluoroquinolone *N*-acetyltransferase AAC(6′)-Ib-cr (9). Plasmid-mediated quinolone resistance (PMQR) is known to involve *aac*(*6′*)*-Ib-cr*, *qnr* family genes, and genes encoding efflux pumps (*qepA* and *oqxAB*) (10). The prevalence of *qnr* and *aac*(*6′*)*-Ib-cr* genes in plasmids of clinical and environmental isolates has been reported worldwide, including in Korea (11, 12). Qnr family proteins interact with their target proteins, thus blocking the action of fluoroquinolones and reducing their inhibitory effect (8). AAC(6′)-Ib-cr is an aminoglycoside acetyltransferase variant with two amino acid substitutions (W102R and D179Y) that confer extended substrate specificity for fluoroquinolones, thus leading to resistance (9).

Members of the genus *Aeromonas* are known to be autochthonous to aquatic environments, although they have been isolated from a wide variety of habitats (13). Some *Aeromonas* species have been recognized as opportunistic human pathogens, as well as primary fish pathogens (13); these species exhibited multidrug resistance, including to fluoroquinolones (14–16). The presence of quinolone resistance determinants has been reported in various *Aeromonas* spp.; several strains contain multiple resistance mechanisms, including mutations at quinolone resistance-determining regions (QRDRs) (17–21), efflux pumps (18, 21, 22), and PMQR genes (23–26). In some *Aeromonas* sp. plasmids, *aac*(*6′*)-*Ib-cr* and *qnrS2* are colocalized with other resistance genes (26–28). Comparative analysis of IncU-type plasmids from *Aeromonas* spp. revealed the conservation of PMQR genes, as well as high genetic plasticity in the region of PMQR genes (26).

In this study, *Aeromonas* sp. strain C3, which displays high-level fluoroquinolone resistance, was isolated from a WWTP and the multiple resistance mechanisms, including PMQR, were characterized in this strain. Comparative analysis of the plasmid from strain C3 revealed a novel mobile insertion cassette element (MICE) related to transposition. The role of the plasmid and the regulation of PMQR in a recipient *Escherichia coli* strain were elucidated by proteome analysis.

## RESULTS

### Isolation and identification of a bacterium with high-level fluoroquinolone resistance.

A bacterial strain isolated from a WWTP displayed unusually high-level resistance to various fluoroquinolones (Table 1). The strain was able to transform fluoroquinolones to their *N*-acetylated metabolites, which were structurally identified by liquid chromatography (LC)-tandem mass spectrometry (MS/MS) and proton nuclear magnetic resonance (NMR) analyses (see Tables S1 and S2) (29). On the basis of its 16S rRNA gene sequence, the isolate showed 99.9% similarity with *Aeromonas hydrophila* subsp. *hydrophila* ATCC 7966^T^. We therefore designated the isolate *Aeromonas* sp. strain C3.

**TABLE 1:** Fluoroquinolone susceptibilities and *N*-acetylation activities of *Aeromonas* sp. strain C3, *E. coli* DH5α, and *E. coli*(pAC3)

Strain and parameter	Efflux inhibitor (concn [mg/liter])	Norfloxacin	Ciprofloxacin	Sarafloxacin	Enrofloxacin	Pefloxacin
*Aeromonas* sp. strain C3						
MIC^a^	None	256	128	128	32	128
MIC	PAβN (80)	256	128	128	16	128
MIC	PAβN (160)	256	128	64	8	64
MIC	NMP (80)	64	32	64	16	64
MIC	NMP (160)	16	16	16	4	32
Activity^b^		13.52 ± 1.40	15.97 ± 3.42	9.13 ± 2.36	ND^c^	ND
*E. coli* DH5α						
MIC	None	0.12	0.01	0.06	0.03	0.25
Activity		ND	ND	ND	ND	ND
*E. coli*(pAC3)						
MIC	None	8	2	8	1	4
Activity		4.59 ± 0.73	5.02 ± 0.09	3.27 ± 0.21	ND	ND

a MICs are expressed in mg/liter.

b Average *N*-acetyltransferase activity ± the standard deviation is expressed in mU/mg of protein.

c ND, not detected.

10.1128/mSphere.00203-17.4 TABLE S1  LC-MS/MS analysis of fluoroquinolones and their *N*-acetylated metabolites. Download TABLE S1, PDF file, 0.1 MB. Copyright © 2017 Kim et al. 2017 Kim et al. This content is distributed under the terms of the Creative Commons Attribution 4.0 International license.

10.1128/mSphere.00203-17.5 TABLE S2  Proton NMR spectral parameters of ciprofloxacin and *N*-acetyl ciprofloxacin. Multiplicities are abbreviated as follows: s, singlet; d, doublet; m, multiplet. Download TABLE S2, PDF file, 0.1 MB. Copyright © 2017 Kim et al. 2017 Kim et al. This content is distributed under the terms of the Creative Commons Attribution 4.0 International license.

### The presence of multiple fluoroquinolone resistance mechanisms in strain C3.

To elucidate this high-level quinolone resistance, the resistance determinants present in the strain were characterized. QRDR sequence analysis showed that point mutations related to resistance were found in the QRDRs of GyrA (D87N) and ParC (S80I). Considering the MICs of *Aeromonas* spp. harboring these mutations (20), the extremely high level of fluoroquinolone resistance exhibited by strain C3 suggested the presence of additional resistance mechanisms. Of the PMQR genes tested, only *aac*(*6′*)-*Ib-cr* and *qnrS* were detected by PCR; these two genes were found to be located in plasmid pAC3 of the strain. The fluoroquinolone resistance of strain C3 was also examined by monitoring changes in MICs in the presence of the efflux pump inhibitors PAβN and NMP, which are known to inhibit a broad range of efflux pumps (30, 31). NMP caused a significant reduction (4- to 16-fold) in the MICs of all of the fluoroquinolones tested (Table 1), while PAβN resulted in minor effects on the MICs of enrofloxacin, sarafloxacin, and pefloxacin.

### Comparative analysis of plasmid pAC3.

**FIG 1:**
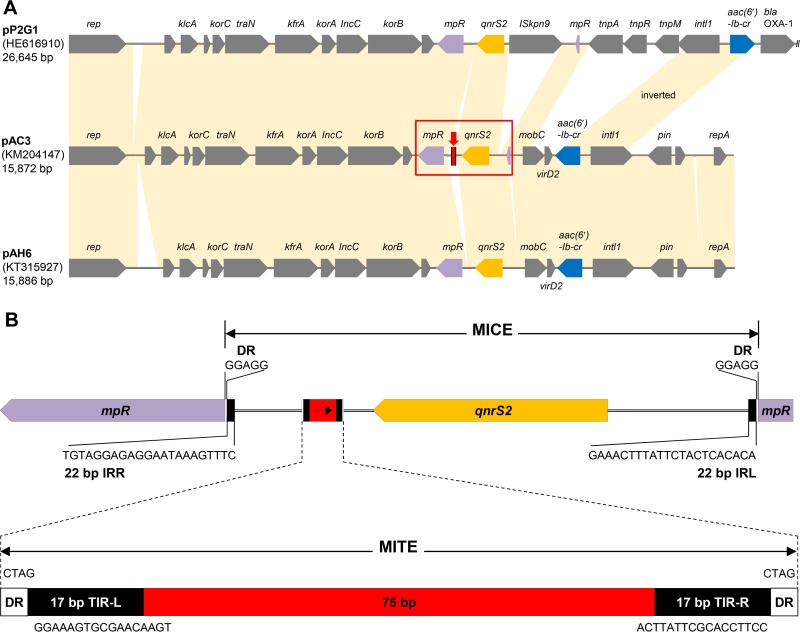
Comparative analysis of *Aeromonas* sp. IncU-type plasmids and the MICE of plasmid pAC3 containing a MITE. (A) Plasmids pAH6 and pP2G1 were used for comparative analysis with plasmid pAC3. Shaded connections between the plasmids show the conserved and shared regions (>99% identity). The red box and arrow indicate the MICE and the MITE, respectively. (B) Genetic map of the MICE region carrying the MITE sequence in plasmid pAC3. IRR, right inverted repeat; IRL, left inverted repeat.

The plasmid harboring the PMQR genes, designated pAC3, was isolated, sequenced, and annotated. The total length of the complete plasmid sequence is 15,872 bp and includes 21 protein-coding genes (Fig. 1A). The plasmid was identified as a member of the IncU-type plasmid family and contained two PMQR genes, *aac*(*6′*)-*Ib-cr* and *qnrS2* (Fig. 1A). For comparative plasmid analyses, the six IncU-type plasmids phylogenetically closest to plasmid pAC3 were selected (see Fig. S1 in the supplemental material) and local colinear block (LCB) analysis was performed with MAUVE software (see Fig. S1). Plasmid pAC3 exhibited the closest relationship with plasmids pAH6 and pP2G1, which possess the identical PMQR genes (Fig. 1A). These plasmids have the same backbone structure, from the replication gene *rep* to the metallopeptidase gene *mpR* (Fig. 1A) (26).

10.1128/mSphere.00203-17.1 FIG S1  Phylogenetic and LCB analyses of IncU-type plasmids. The phylogenetic tree shown was constructed with MEGA 6.0, and LCB analysis was performed with MAUVE software. IncU-type plasmids, including pAC3, pAH6, pP2G1, pRA3, pFBAOT6, pAH227, and pASCH21, were used for analyses. IncQ-type plasmid pRAS3.1 was used as an outgroup. Download FIG S1, PDF file, 0.4 MB. Copyright © 2017 Kim et al. 2017 Kim et al. This content is distributed under the terms of the Creative Commons Attribution 4.0 International license.

A MICE bracketed by 22-bp left and right inverted repeats was found to contain the *qnrS2* gene. Insertion of the MICE disrupted the *mpR* gene encoding a putative zinc metalloprotease. IncU plasmids carrying the *qnrS2* gene from other *Aeromonas* spp. have been found in Europe and Asia (26–28). The presence of the *qnrS2*-carrying MICE structure in different *Aeromonas* species from geographically distant aquatic environments suggests that this PMQR determinant is widespread in this genus, and these MICE-type structures are potential vehicles of PMQR determinants (32). Interestingly, a novel genetic structure not present in other MICE structures found in *Aeromonas* species (26–28) was discovered inside the MICE of plasmid pAC3 and exhibited a typical miniature inverted-repeat transposable element (MITE) structure (33, 34). The MITE consists of a 75-bp core sequence bracketed by 4-bp direct repeats (DRs) and 17-bp terminal inverted repeats (TIRs) (Fig. 1B). Both the MICE and the MITE are nonautonomous derivatives of insertion sequences generated by internal deletion, and the difference between these two elements is the presence of coding sequences (CDSs) (35). In the present study, the MICE harbored the *qnrS2* gene while the MITE carried no passenger gene.

**FIG 2:**
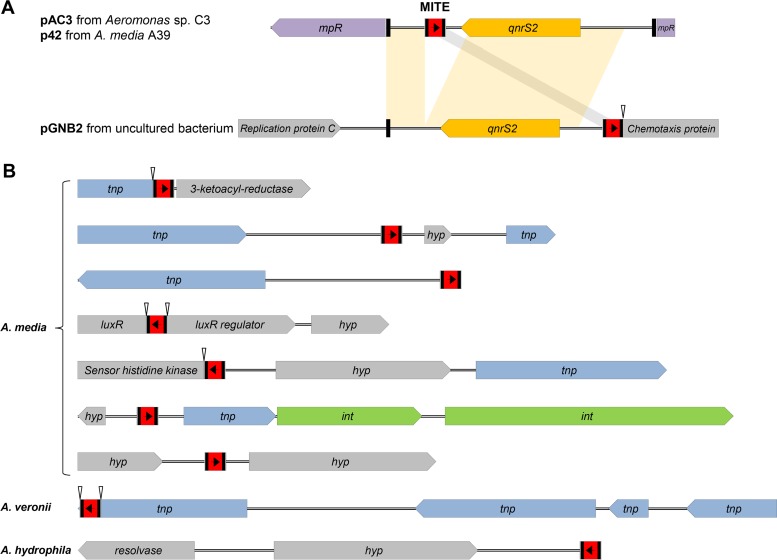
Genetic characterization of the MITE-harboring regions in *Aeromonas* sp. plasmids and chromosomes. (A) Comparison of plasmids p42 and pGNB2 with plasmid pAC3. Shaded connections between the plasmids show the conserved and shared regions. (B) Genetic map of regions containing MITE sequences in *Aeromonas* sp. chromosomes. White arrowheads indicate gene truncations; the transposase-, integrase-, and hypothetical-protein-encoding genes are *tnp*, *int*, and *hyp*, respectively.

MITE sequences have not been previously identified in *Aeromonas* species. The present study revealed that these sequences are also present in the partial fragment sequence of plasmid p42 from *A. media* A39 (24); pGNB2 from an uncultured bacterium (36); and the chromosomes of *A. media* WS, *A. veronii* AVNIH1, and *A. hydrophila* GYK1. The MITE of plasmid p42 is identical to that of plasmid pAC3; in contrast to plasmid pAC3, in IncQ-type plasmid pGNB2, the MITE is found upstream of the *qnrS2* gene (Fig. 2A). The MITE sequences were also frequently found in the genome of *A. media* WS (Fig. 2B). All of these MITE sequences were highly conserved, except for the DRs (see Fig. S2).

10.1128/mSphere.00203-17.2 FIG S2  Alignment of multiple MITE sequences from plasmids pAC3, p42, and pGNB2, as well as the genomes of *A. media* WS, *A. veronii* AVNIH1, and *A. hydrophila* GYK1. Light gray-, dark gray-, and red-shaded sequences indicate 4-bp DR, 17-bp TIR, and 75-bp core sequences of the MITE, respectively. Download FIG S2, PDF file, 0.1 MB. Copyright © 2017 Kim et al. 2017 Kim et al. This content is distributed under the terms of the Creative Commons Attribution 4.0 International license.

### The role of PMQR proteins in fluoroquinolone resistance.

Introduction of PMQR into other bacteria poses a potential risk of dissemination of antibiotic resistance. Plasmid pAC3 was successfully introduced into antibiotic-susceptible *E. coli* DH5α. Transformants cultured in the presence of fluoroquinolones showed the production of *N*-acetylated fluoroquinolones. To further elucidate the resistance mechanisms of PMQR, an antibiotic susceptibility test and an *N*-acetylation activity assay against five different fluoroquinolones were performed with *Aeromonas* sp. strain C3, parental *E. coli* DH5α, and *E. coli* DH5α harboring plasmid pAC3 [*E. coli*(pAC3)]. The results demonstrate that *E. coli*(pAC3) acquired resistance to all of the fluoroquinolones tested (Table 1). Similar to that from strain C3, the cell-free protein extract from *E. coli*(pAC3) was also able to transform norfloxacin, ciprofloxacin, and sarafloxacin into their *N*-acetylated metabolites (Table 1). No activity was detected with enrofloxacin and pefloxacin, which are structurally inaccessible to AAC(6′)-Ib-cr. The acetyltransferase activity present in *E. coli*(pAC3) suggests that the introduced resistance was conferred by AAC(6′)-Ib-cr. Interestingly, the MICs of enrofloxacin and pefloxacin for *E. coli*(pAC3) increased significantly, although acetyltransferase activity was not detected with these substrates. These results imply that *qnrS2*, which is also located on the plasmid, contributes to resistance.

### Expression of PMQR proteins.

**FIG 3:**
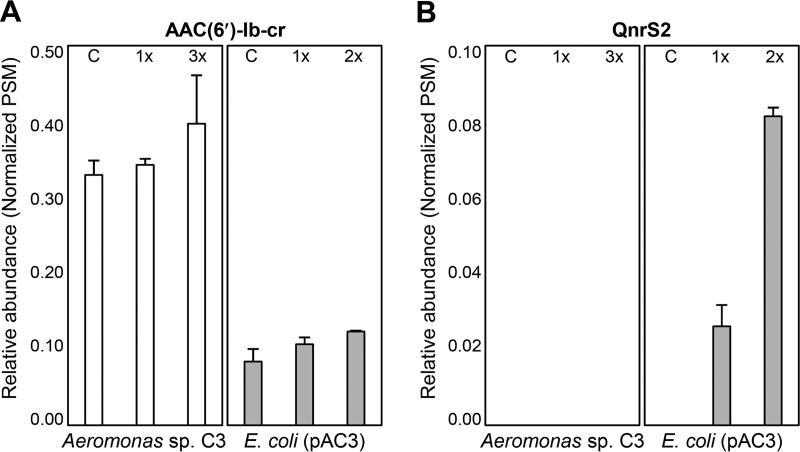
Expression of AAC(6′)-Ib-cr (A) and QnrS2 (B) in *Aeromonas* sp. strain C3 and *E. coli*(pAC3). C, 1×, 2×, and 3× indicate 0-, 1-, 2-, and 3-fold MIC fluoroquinolone treatments, respectively. Protein expression levels are expressed as normalized peptide spectrum matches (PSMs).

To further confirm the role of the PMQR of plasmid pAC3, the expression of AAC(6′)-Ib-cr and QnrS2 was analyzed by a proteomics approach. Proteomes were analyzed by using cells spiked at mid-exponential phase with fluoroquinolones at concentrations based on the MICs (see Table S3). The numbers of proteins detected by proteome analysis are detailed in Table S3. Proteomes obtained from strain C3, *E. coli* DH5α, and *E. coli*(pAC3) cells treated with ciprofloxacin (at the MIC) and enrofloxacin (two or three times the MIC) were compared with those of untreated cells. AAC(6′)-Ib-cr was found to be constitutively expressed in both strain C3 and *E. coli*(pAC3) regardless of fluoroquinolone treatment and the host cell (Fig. 3A). The level of AAC(6′)-Ib-cr expression in strain C3 was 3- to 4-fold higher than that in *E. coli*(pAC3) (Fig. 3A). These results coincide with the higher *N*-acetylation activities of the cell-free protein extract from strain C3 than that from *E. coli*(pAC3) (Table 1). QnrS2 was expressed in *E. coli*(pAC3) when the cells were treated with fluoroquinolones (Fig. 3B). These results explain why *E. coli*(pAC3) acquired resistance to enrofloxacin and pefloxacin (Table 1). In contrast, QnrS2 expression was not detected in strain C3 under the conditions used (Fig. 3B); QnrS2 was not expressed, even under higher concentrations of fluoroquinolone treatment (up to 16 and 32 times the MIC), suggesting that it may not be significantly involved in fluoroquinolone resistance in the *Aeromonas* strain.

10.1128/mSphere.00203-17.6 TABLE S3  Summary of proteome results. Download TABLE S3, PDF file, 0.1 MB. Copyright © 2017 Kim et al. 2017 Kim et al. This content is distributed under the terms of the Creative Commons Attribution 4.0 International license.

### The role of plasmid pAC3 in recipient cells in response to fluoroquinolone.

A global proteome analysis revealed that a fluoroquinolone target protein (GyrA) was upregulated in response to antibiotic treatment in *E. coli* DH5α and to a lesser degree in *E. coli*(pAC3) (see Fig. S3), suggesting that the presence of the plasmid might affect GyrA expression. In addition, expression of AcnB, which is known to be involved in cellular death induced by bactericidal antibiotics (37), increased in response to fluoroquinolone treatment in *E. coli* DH5α but remained relatively constant in *E. coli*(pAC3) (see Fig. S3). The expression of several major regulators, including AcrA, IhfA, Crp, HNS, and LRP, decreased in response to fluoroquinolone treatment in *E. coli* DH5α and to a lesser extent in *E. coli*(pAC3) (see Fig. S3). These results suggest that the presence of the plasmid in recipient cells may contribute to the reduction of antibiotic stress.

10.1128/mSphere.00203-17.3 FIG S3  Expression of fluoroquinolone-induced proteins (A) and major regulators (B) in *E. coli* DH5α and *E. coli*(pAC3) in response to fluoroquinolone treatment. C, 1×, and 2× indicate 0-, 1-, and 2-fold MIC fluoroquinolone treatments, respectively. Protein expression levels are expressed as normalized peptide spectrum matches (PSMs). NrdA, ribonucleoside-diphosphate reductase 1 alpha subunit; NrdB, ribonucleoside-diphosphate reductase 1 beta subunit; RecA, DNA recombination and repair protein; GyrA, DNA gyrase subunit A; AcnB, aconitase B; ArcA, response regulator of two-component regulatory system; IhfA, integration host factor DNA-binding protein; Crp, cyclic-AMP-activated global transcription factor; HNS, global DNA-binding transcriptional dual regulator; LRP, DNA-binding transcriptional dual regulator. Download FIG S3, PDF file, 0.1 MB. Copyright © 2017 Kim et al. 2017 Kim et al. This content is distributed under the terms of the Creative Commons Attribution 4.0 International license.

### Regulation of QnrS2 expression in recipient cells.

**FIG 4:**
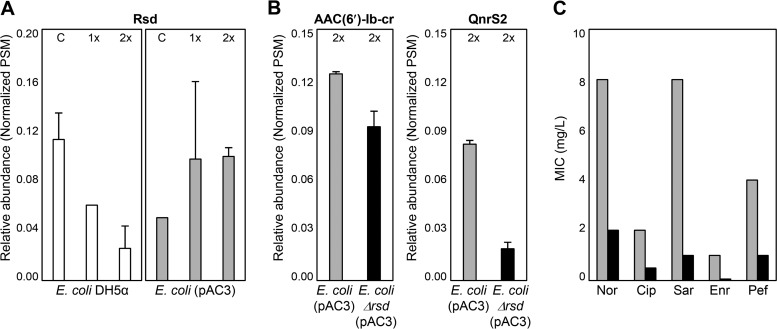
Differential expression of Rsd (A) and PMQR (B) proteins in *E. coli* strains and susceptibility of the strains to various fluoroquinolones (C). White, gray, and black bars indicate *E. coli* DH5α, *E. coli*(pAC3), and *E. coli* Δrsd(pAC3), respectively. C, 1×, and 2× indicate fluoroquinolone treatments at 0-, 1-, and 2 times the MIC, respectively. Protein expression levels are expressed as normalized relative peptide spectrum matches (PSMs). Nor, Cip, Sar, Enr, and Pef indicate norfloxacin, ciprofloxacin, sarafloxacin, and pefloxacin, respectively.

While fluoroquinolone treatment increased the expression of QnrS2 in *E. coli*(pAC3) (Fig. 3B), the expression of ribonucleoside-diphosphate reductase (NrdA and NrdB) also showed a fluoroquinolone-induced increase in both *E. coli* DH5α and *E. coli*(pAC3) (see Fig. S3). The expression of SOS response regulator protein RecA did not increase under these conditions (see Fig. S3). It has been previously reported that NrdA and NrdB were upregulated by fluoroquinolone treatment (38) and that their expression was independent of the SOS response (39). Our results also suggest that expression of QnrS2 was induced not by the SOS response but rather by fluoroquinolone-dependent signaling, as previously reported (40–42). In addition to the major regulators examined, the expression of regulator of sigma D (Rsd) also decreased in response to fluoroquinolone in *E. coli* DH5α (Fig. 4A). However, *rsd* expression was upregulated in *E. coli*(pAC3) (Fig. 4A), suggesting that *rsd* may be associated with QnrS2 expression. The proteome of an *E. coli rsd* deletion mutant harboring pAC3 [*E. coli* Δrsd(pAC3)] in response to fluoroquinolone was compared with that of *E. coli*(pAC3). While the expression of AAC(6′)-Ib-cr did not vary greatly, the expression of QnrS2 was significantly lower in *E. coli* Δrsd(pAC3) than in *E. coli*(pAC3) when the strains were treated with the same amount of antibiotic stress (twice the MIC) (Fig. 4B). Furthermore, the *rsd* deletion mutant was much more susceptible than *E. coli*(pAC3), with approximately 4-fold lower MICs than those of *E. coli*(pAC3) (Fig. 4C); although its MICs were higher than those of *E. coli* DH5α (Table 1; Fig. 4C). These results suggest that the *rsd* gene plays a role in resistance via the regulation of QnrS2 expression.

## DISCUSSION

Fluoroquinolone-resistant *Aeromonas* spp. have been identified in both environmental and clinical isolates; in several strains, high-level fluoroquinolone resistance was found to be conferred by multiple resistance mechanisms (16, 18, 23). In the present study, *Aeromonas* sp. strain C3, which was highly resistant to fluoroquinolones, was isolated from a WWTP and found to possess mutations in *gyrA* and *parC*. Efflux pumps were also shown to contribute to resistance, as previously reported in this genus (18, 21, 22). The presence of 10 resistance-nodulation-division efflux systems in the genome of *A. hydrophila* subsp. *hydrophila* ATCC 7966 (22, 43) explains the complex changes in resistance to various fluoroquinolones in the presence of efflux inhibitors (Table 1).

Sequencing analysis of plasmid pAC3 present in strain C3 identified two PMQR determinants, *qnrS2* and *aac*(*6′*)*-Ib-cr*. Recent comparative analysis studies of *Aeromonas* sp. plasmids belonging to the same incompatibility group have revealed the conservation and variation in their sequences and genetic structures (26, 44). In the present study, we identified a mobile insertion cassette with a unique structure containing the PMQR gene *qnrS2* and a novel MITE structure. MITEs are small, nonautonomous mobile elements broadly dispersed in prokaryotes, although they have been formalized as nonautonomous transposable sequences in plants (34). Bacterial MITE sequences are primarily found in intergenic regions of the chromosome; however, they are also present intragenically (34). In this study, a MITE structure was identified within the MICE inserted in the *mpR* gene in plasmid pAC3. The MITE sequence also showed typical signatures of TIRs, a target site duplication consisting of DRs and a core sequence lacking a transposase gene. However, in this study we demonstrate a plasmid location for the MITE associated with an antibiotic resistance gene (ARG). There have been only a few reports on ARG-associated MITEs. A special type of MITE, termed an integron mobilization unit, has been identified in the *bla*_GES-5_-carrying plasmid of carbapenem-resistant *Enterobacter cloacae* (45). MITE-flanked integrons carrying ARGs were also revealed in several *Acinetobacter* strains (46, 47). These studies suggested that MITEs might be associated with the mobilization of ARGs. The MITE structures in the present study are the first characterized in an *Aeromonas* species (Fig. 2). The frequent occurrence of MITE structures in *Aeromonas* spp. suggests that they play a role in the evolution of this genus (Fig. 2B). In particular, the MITE sequences in *A. media* and *A. veronii* are associated with nearby transposases (Fig. 2B), raising the possibility of transposition.

Comparative analysis of the proteomes of *E. coli* DH5α and its derivative harboring plasmid pAC3 revealed constitutive expression of AAC(6′)-Ib-cr and fluoroquinolone-dependent expression of QnrS2 in the recipient strain. The proteome results also suggest that the presence of the plasmid in the recipient strain may reduce antibiotic stress through the expression of PMQR proteins, thus influencing the cellular regulatory network. The promoter sequence of *aac*(*6′*)*-Ib-cr* in pAC3 was found to be conserved with those from many other bacteria, indicating that constitutive and host-independent expression of *aac*(*6′*)*-Ib-cr* could be a general feature. Differential expression of QnrS1 has been reported in a fluoroquinolone-sensitive *E. coli* strain and a fluoroquinolone-resistant *E. coli* strain harboring a mutated *gyrA* gene (41). In the present study, *rsd* in the recipient strain was shown to be involved in antibiotic stress-dependent expression of *qnrS2*, independent of the SOS response. Although *qnrS2* has been identified as a fluoroquinolone resistance determinant in many *Aeromonas* species (48, 49), there is no direct evidence that the gene is actually expressed in *Aeromonas*. A previous study has reported that *A. allosaccharophila*, which also contains the two PMQR determinants [*qnrS2* and *aac*(*6′*)*-Ib-cr*] on a plasmid, remained susceptible to quinolones and that these genes might spread silently (27). The putative promoter region of *qnrS2* was highly conserved in the plasmids of *Aeromonas* spp., indicating that these features may be conserved in this genus. It is still unclear whether the regulation system of *qnrS2* expression in *Aeromonas* is functional.

To the best of our knowledge, this is the first report of a novel mobile insertion cassette structure in the PMQR region of an environmental *Aeromonas* species, suggesting that the mobile element may play an important role in *qnrS2* dissemination. The present study also demonstrates that the fluoroquinolone-dependent expression of *qnrS2* is associated with *rsd* in *E. coli* DH5α harboring plasmid pAC3. Our results suggest that the genus *Aeromonas* is important as a reservoir of fluoroquinolone resistance determinants in the environment and should be under intensive surveillance for antibiotic resistance.

## MATERIALS AND METHODS

### Chemicals and media.

Ciprofloxacin, norfloxacin, enrofloxacin, phenylalanine arginine β-naphthylamide (PAβN), and 1-(1-naphthylmethyl)-piperazine (NMP) were purchased from Sigma-Aldrich (St. Louis, MO). Pefloxacin and sarafloxacin were obtained from Santa Cruz Biotechnology (Dallas, TX). R2A, Luria-Bertani (LB), and Mueller-Hinton (MH) media were purchased from BD Science (San Jose, CA).

### Isolation and identification of the bacterial strain.

A sludge sample obtained from a WWTP in Anseong, South Korea, was inoculated onto R2A agar supplemented with 100 mg/liter ciprofloxacin and incubated at 30°C for 48 h to obtain bacteria with high-level fluoroquinolone resistance. The 16S rRNA gene of the isolate was amplified and sequenced with universal primers (27F and 1492R) and identified by using the EzTaxon-e database (http://www.ezbiocloud.net/) (50).

### Analysis of antimicrobial susceptibility.

MICs of fluoroquinolones were determined by the broth microdilution method detailed in the Clinical and Laboratory Standards Institute (CLSI) guidelines (51). Susceptibility of the strain to fluoroquinolones was assessed in the absence or presence of PAβN or NMP (efflux pump inhibitors) as previously described (30, 31).

### Characterization of fluoroquinolone resistance genes.

The presence of fluoroquinolone resistance genes, including *qnrA*, *qnrB*, *qnrC*, *qnrD*, *qnrS*, *qnrVC1*, *qnrVC4*, *qepA*, *aac*(*6′*)*-Ib-cr*, *oqxA*, and *oqxB* (49, 52–54), and mutations in the QRDRs of the *gyrA*, *gyrB*, and *parC* genes (17, 18) were verified by PCR amplification and sequencing as previously described (see Table S4).

10.1128/mSphere.00203-17.7 TABLE S4  PCR primers used in this study. Download TABLE S4, PDF file, 0.1 MB. Copyright © 2017 Kim et al. 2017 Kim et al. This content is distributed under the terms of the Creative Commons Attribution 4.0 International license.

### Enzyme assay.

Bacterial strains were cultivated in LB medium at 30°C until cultures reached the mid-exponential growth phase. Cells were harvested 3 h following the addition of ciprofloxacin at a concentration of 20 mg/liter. Cells were harvested and disrupted by sonication in 20 mM Tris-HCl buffer (pH 7.5), and cell debris was removed by filtration (0.2 μm) to obtain cell-free protein extracts. The reaction mixture, consisting of 90 µM fluoroquinolone substrate, 200 µM acetyl coenzyme A, and 50 to 100 µg of protein extract in 500 µl of 50 mM Tris-HCl buffer (pH 7.5), was incubated at 30°C. Samples taken from the reaction mixtures were subjected to high-performance liquid chromatography (HPLC) analysis with an Atlantis dc18 column (4.6 by 250 mm; Waters Corp., Milford, MA) and a Varian ProStar HPLC system (Varian, Inc., Walnut Creek, CA) set at 280 nm in a diode array detector. The mobile phase consisted of a linear gradient of acetonitrile (10 to 95%) containing 0.1% formic acid at a flow rate of 1 ml/min. One enzyme unit was defined as the amount of enzyme required to convert 1 μmol of substrate to its *N*-acetylated product per minute at 30°C.

### Genetic manipulation of *E. coli* strains.

Plasmid pAC3 was introduced into fluoroquinolone-sensitive *E. coli* strain DH5α by the transformation method (55), and transformants were selected in the presence of norfloxacin (1 mg/liter). Precise deletion-replacement of the *rsd* gene from *E. coli* was conducted by the method of Datsenko and Wanner (56) to obtain *E. coli* Δrsd.

### Sequencing and comparative analysis of the plasmid.

The plasmid was isolated by the maxipreparation method as previously described (57). The purified plasmid was treated with Plasmid-Safe ATP-Dependent DNase (Epicentre, Madison, WI) to remove chromosomal DNA contaminants. The plasmid DNA was fragmented with dsDNA Fragmentase (NEB, Hitchin, United Kingdom) to make a proper size for library construction. The DNA fragments were processed with the TruSeq DNA sample preparation kit v 2 (Illumina, San Diego, CA) in accordance with the manufacturer’s instructions. The library was quantified with a Bioanalyzer 2100 (Agilent Technologies, Santa Clara, CA), and the average size of the library was 300 bp. Plasmid pAC3 was sequenced with the Illumina MiSeq platform (Illumina) at Chunlab (Seoul, South Korea). The paired-end sequencing reads generated were assembled with CLC genomics workbench 6.0 (CLC Bio, Boston, MA), and the contigs and PCR-based gap reads were combined with CodonCode aligner 3.7.1 (CodonCode Corp., Centerville, MA). The CDSs were predicted by Glimmer 3.02 (58). For functional annotation, the predicted CDSs were compared to those previously deposited with catalytic families (CatFam), the COG database, NCBI reference sequences (RefSeq), and the SEED subsystem (59–62). Comparison of plasmid pAC3 with *Aeromonas* sp. plasmids was conducted by phylogenetic and LCB analyses as previously described (see Fig. S1) (44). Plasmids pAH6 and pP2G1 were used for the comparative analysis of IncU-type plasmids. A partial fragment sequence of plasmid p42; the complete sequence of plasmid pGNB2; and the genome sequences of *A. media* WS, *A. veronii* AVNIH1, and *A. hydrophila* GYK1 were used for comparison of transposable elements with plasmid pAC3.

### Proteome analysis.

Cells of *Aeromonas* sp. strain C3, *E. coli* DH5α, and derivative *E. coli* strains were prepared as described above. Ciprofloxacin and enrofloxacin were added to each strain at the MIC to three times the MIC. Cells were suspended in lysis buffer (50 mM Tris-HCl buffer [pH 7.8], 6 M guanidine hydrochloride, 10 mM dithiothreitol), and cell-free protein extracts were obtained by sonication. The protein samples were reduced, alkylated, and precipitated with dithiothreitol (10 mM), iodoacetamide (100 mM), and trichloroacetic acid (30%, wt/vol), respectively. The dried protein pellets were dissolved in 50 mM ammonium bicarbonate and digested with trypsin (Thermo Scientific, Waltham, MA). The trypsin digests were cleaned up with Sep-Pak C_18_ columns (Waters Corp., Milford, MA). One microgram of sample in 0.4% acetic acid was loaded onto a linear ion trap mass spectrometer (LTQ Velos; Thermo Scientific) coupled with a nanosprayer (Thermo Scientific) and a nano column (8.5 cm by 75 μm) packed with C_18_ medium (200 Å Magic; Michrom Bioresources, Auburn, CA) (63). The organic mobile phase consisted of a linear gradient of acetonitrile (5 to 30%) containing 0.1% formic acid for 380 min at a flow rate of 70 μl/min. The mass spectrometer was operated with the following parameters and options: a 2.0-kV nanospray distal voltage, a capillary temperature of 200°C, and full-scan mode (300 to 5,000 Da). MS/MS data were acquired and deconvoluted with Xcalibur 2.1 (Thermo Scientific), and the whole data set was searched with the SEQUEST search algorithm (64) implemented in the Proteome Discoverer 1.3 software (Thermo Scientific). For protein identification, the genome sequences of *E. coli* K-12 strain MG1655 (accession no. NC_000913) and plasmid pAC3 (accession no. KM204147) were used as databases. The genome of *A. hydrophila* subsp. *hydrophila* ATCC 7966 (accession no. CP000462) was used as a reference genome for quantification of the relative abundance of plasmid proteins in strain C3. Filter parameters for peptide identification (medium peptide confidence [ΔCn] of >0.1 and false discovery rate of <5%) and protein identification (more than two peptides per protein) were applied to the spectra searched by SEQUEST. The shared proteome of biological duplicate samples was used for further analysis, and the protein expression level was determined by using normalized spectral counts.

### Accession number(s).

The nucleotide sequence of plasmid pAC3 was deposited in GenBank (http://www.ncbi.nlm.nih.gov/GenBank) under accession number KM204147. The accession numbers of the other plasmid and genome sequences used in the study are as follows: pAH6, KT315927; pP2G1, HE616910; p42, EU439941; pGNB2, DQ460733; *A. media* WS, CP007567; *A. veronii* AVNIH1, CP014774; *A. hydrophila* GYK1, CP016392.
