# Supplementary material for: Unique Features of Aeromonas Plasmid pAC3 and Expression of the Plasmid-Mediated Quinolone Resistance Genes
Source: mSphere. 2017 May 24;2(3):e00203-17. doi: 10.1128/mSphere.00203-17 (PMC5444012; doi:10.1128/mSphere.00203-17)
Supplement: TABLE S1 [file sph003172292st4.pdf]

Compound	UV λ_{max} (nm)	Parent-ion $[M+H]^+$ m/z	Product-ion spectra (m/z) with relative abundance (%)
Ciprofloxacin	280	332	314(75), 288(100), 268(100), 245(72), 231(20), 277(8), 205(15)
N-Acetylciprofloxacin	283	374	356(100), 314(58), 295(46), 272(55), 253(18), 243(25), 231(100)
Norfloxacin	279	320	302(35), 276(100), 256(100), 233(73), 219(15)
N-Acetylnorfloxacin	283	362	344(100), 316(10), 302(5), 274(100), 231(5)
Sarafloxacin	216, 281	386	368(44), 342(100), 342(15), 322(100), 299(68), 285(14)
N-Acetylsarafloxacin	237, 286	428	410(100), 390(10), 368(8), 325(4), 299(5)
