# Supplementary material for: Unique Features of Aeromonas Plasmid pAC3 and Expression of the Plasmid-Mediated Quinolone Resistance Genes
Source: mSphere. 2017 May 24;2(3):e00203-17. doi: 10.1128/mSphere.00203-17 (PMC5444012; doi:10.1128/mSphere.00203-17)
Supplement: TABLE S2 [file sph003172292st5.pdf]

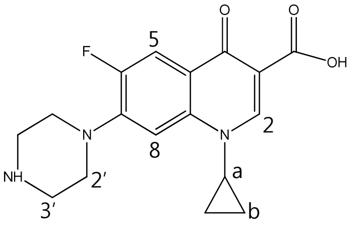

Ciprofloxacin

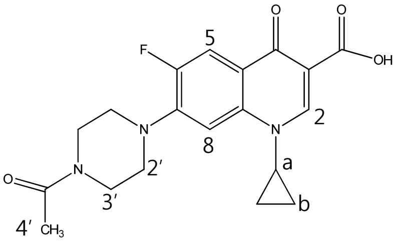

N-Acetylciprofloxacin

Proton	Ciprofloxacin				*N*-Acetylciprofloxacin			
	δ (ppm)	Integral	Mult.*	*J* (Hz)	δ (ppm)	Integral	Mult.*	*J* (Hz)
N-H	-	-	-	-	-	-	-	-
H2	8.73	1.55	s	-	8.74	9.36	s	-
H5	7.98<br>7.95	1.71	d	13.7	8.01<br>7.99	10.60	d	13.34
H8	7.56<br>7.55	1.78	d	7.7	7.58<br>7.57	11.15	d	7.5
H2′	3.01	6.01	m	4.7	3.39	21.24	m	4.8
H2′					3.33	21.08	m	5.1
H3′	3.30	5.80	m	4.6	3.75	21.71	m	4.8
H3′					3.69	20	m	4.4
H4′					2.10	29.88	s	
Ha	3.68	1.83	m		3.69	ca. 10	m	
Hb	1.37<br>1.36	4.15	m	6.0	1.38<br>1.37	23.93	m	6.6
Hb	1.16	3.85	m	7.02	1.17	22.12	m	7.01
