# Supplementary figures and images for: Unique Features of Aeromonas Plasmid pAC3 and Expression of the Plasmid-Mediated Quinolone Resistance Genes
Source: mSphere. 2017 May 24;2(3):e00203-17. doi: 10.1128/mSphere.00203-17 (PMC5444012; doi:10.1128/mSphere.00203-17)

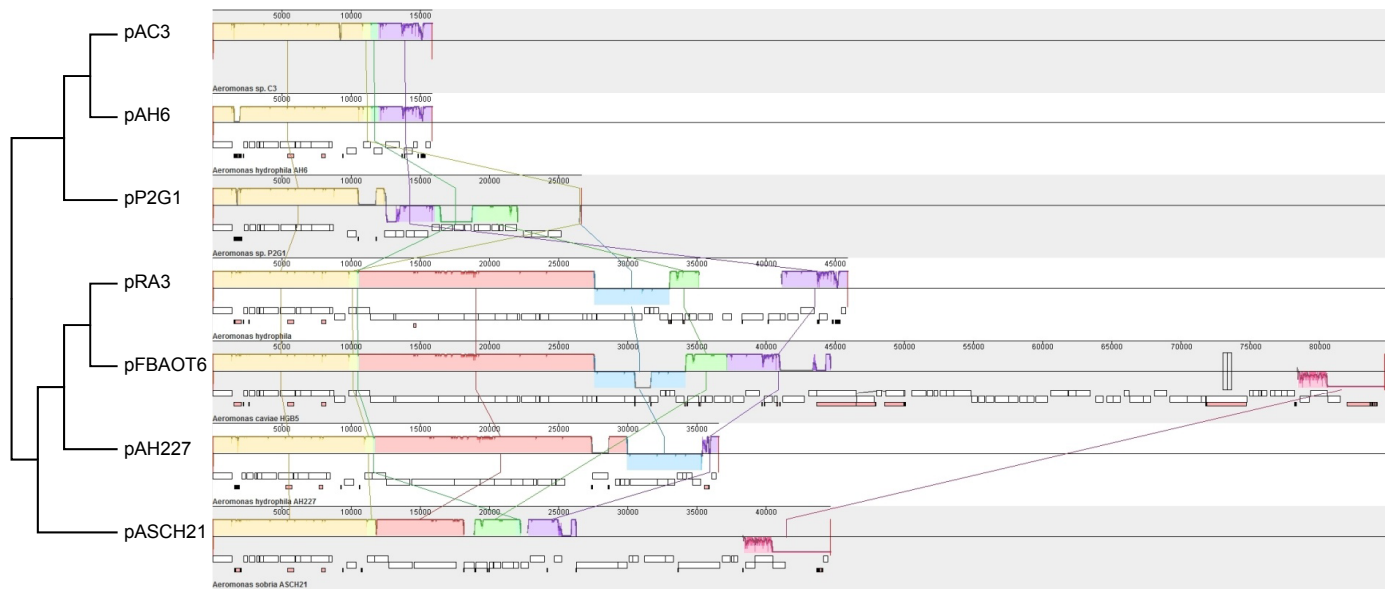

Supplement: FIG S1 [file sph003172292sf1.pdf]

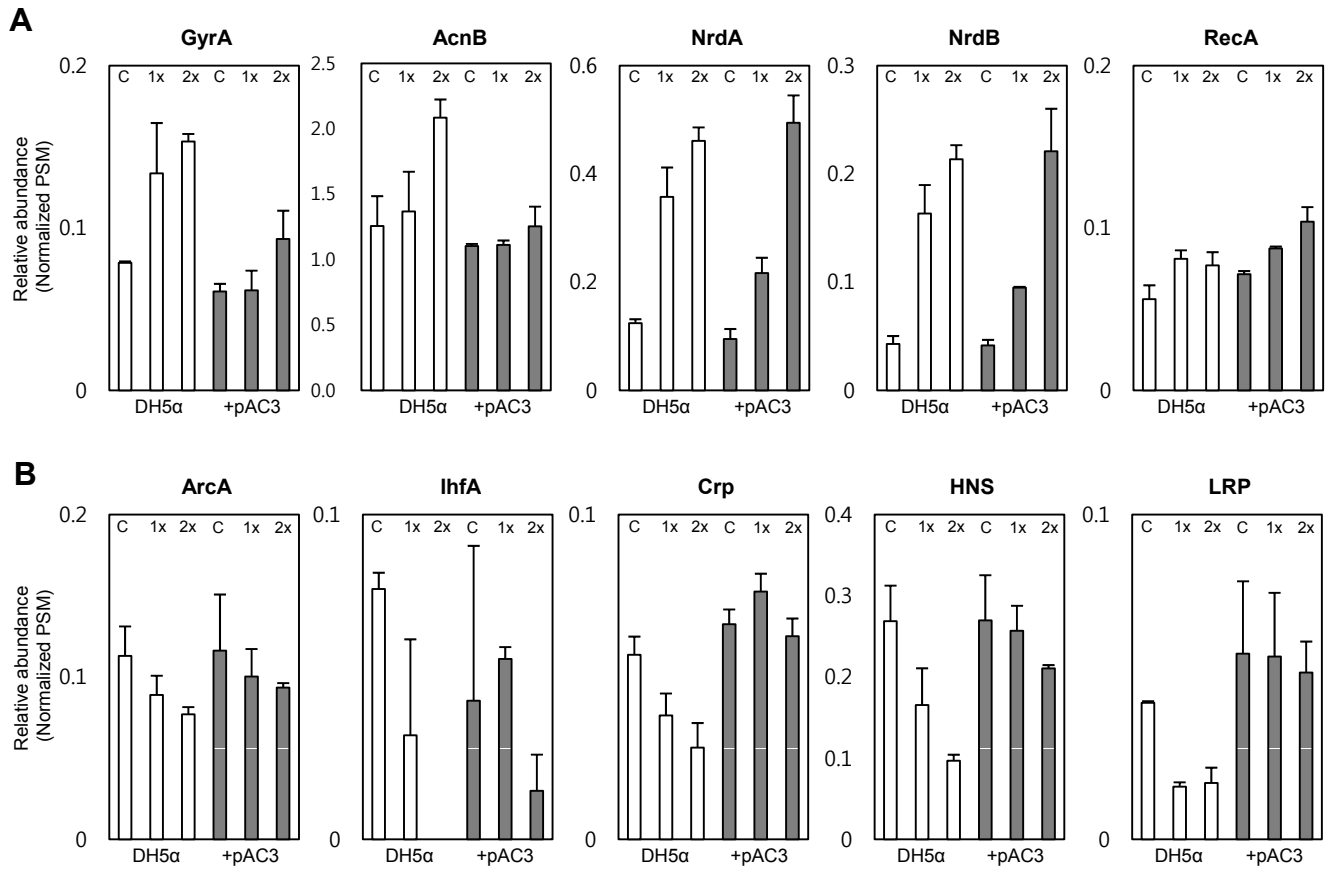

Supplement: FIG S3 [file sph003172292sf3.pdf]
