# Supplementary material for: Unique Features of Aeromonas Plasmid pAC3 and Expression of the Plasmid-Mediated Quinolone Resistance Genes
Source: mSphere. 2017 May 24;2(3):e00203-17. doi: 10.1128/mSphere.00203-17 (PMC5444012; doi:10.1128/mSphere.00203-17)
Supplement: FIG S2 [file sph003172292sf2.pdf]

```
pAC3   CTAGGGAAAGTGCGAACAAGTATCCCAAGAACAAAAAATGAAAATTTTAGTCATTTAATATCAATGGGTTACCAGTAAATGCTCTCGTGCCATATGACTTATTCGCACCTTCCCTAG
p42    CTAGGGAAAGTGCGAACAAGTATCCCAAGAACAAAAAATGAAAATTTTAGTCATTTAATATCAATGGGTTACCAGTAAATGCTCTCGTGCCATATGACTTATTCGCACCTTCCCTAG
pGNB2  TTAGGGAAAGTGCGAACAAGTATCCCAAGAACAAAAAATGAAAATTTTAGTCATTTAATATCAATGGGTTACCAGTGAATGCTCTCGTGCCATATGACTTATTCGCACCTTCCTTAG
WS     TAAGGGAAAGTGCGAACAAGTATCCCACGAACAAAAAATGAAAATTTTAGTCATTTAATATCAATGGGTTACCAGTAAATGCTCTCGTGCCATATGACTTATTCGCACCTTCCTTAG
WS     CTAAGGAAAGTGCGAACAAGTATCCCACGAACAAAAAATGAAAATTTTAGTCATTTAATATCAATGGGTTACCAGTAAATGCTCTCGTGCCATATGACTTATTCGCACCTTCCCCTA
WS     TTAGGGAAAGTGCGAACAAGTATCCCAAGAACAATAAATGAAAATTTTAGTCATTTAATATCAATGGGTTACCAGTAAATGCTCTCGTGCCATATGACTTATTCGCACCTTCCCTAG
WS     CTAAGGAAAGTGCGAACAAGTATCCCAAGAACAAAAAATGAAAATTTTAGTCATTTAATATCAATGGGTTACCAGTAAATGCTCTCGTGCCATATGACTTATTCGCACCTTCCCTAA
WS     CTAAGGAAAGTGCGAACAAGTATCCCACGAACAAAAAATGAAAATTTTAGTCATTTAATATCAATGGGTTACCAGTAAATGCTCTCGTGCCATATGACTTATTCGCACCTTCCCCAA
WS     CTAAGGAAAGTGCGAACAAGTATCCCACGAACAAAAAATGAAAATTTTAGTCATTTAATATCAATGGGTTACCATTAAATGCTCTCGTGCCATATGACTTATTCGCACCTTCCCTAG
WS     CTAGGGAAAGTGCGAACAAGTATCCCACGAACAAAAAATGAAAATTTTAGTCATTTAATATCAATGGGTTACCAGTAAATGCTCTCGTGCCATATGACTTATTCGCACCTTCCCTAA
AVNIH1 CTAAGGAAAGTGCGAACAAGTATCCCACGAACAAAAAATGAAAATTTTAGTCATTTAATATCAATGGGTTACCAGTAAATGCTCTCGTGCCATATGACTTATTCGCACCTTCCCTAG
GYK1   TTAGGGAAAGTGCGAACAAGTATCCCACGAACAAAAAATGAAAATTTTAGTCATTTAATATCAATGGGTTACCAGTAAATGCTCTCGTGCCATATGACTTATTCGCACCTTCCTTAG
```
