# Supplementary material for: Unique Features of Aeromonas Plasmid pAC3 and Expression of the Plasmid-Mediated Quinolone Resistance Genes
Source: mSphere. 2017 May 24;2(3):e00203-17. doi: 10.1128/mSphere.00203-17 (PMC5444012; doi:10.1128/mSphere.00203-17)
Supplement: TABLE S3 [file sph003172292st6.pdf]

Microorganism	Treatment	Replicate	Dose (μg/ml)	Fold for MIC value	No. of PSMs	No. of peptides	No. of Proteins	No. of shared proteins in biological duplicate (Coverage, %)
Aeromonas sp. strain C3	Control	1	0	-	46,189	4,594	770	660 (98.57)
		2	0	-	48,949	4,552	779	
	Ciprofloxacin	1	100	0.8	56,620	4,805	787	622 (97.89)
		2	100	0.8	47,600	3,710	689	
	Enrofloxacin	1	100	3.1	39,491	4,455	787	603 (98.24)
		2	100	3.1	29,618	3,566	668	
	Enrofloxacin	1	512	16	59,291	5293	834	744 (99.11)
		2	512	16	62,405	5479	881	
	Enrofloxacin	1	1,024	32	67,747	5632	885	751 (99.12)
		2	1,024	32	66,186	5,647	869	
E. coli DH5α	Control	1	-	-	48,217	3926	882	822 (99.18)
		2	-	-	48,551	3966	897	
	Ciprofloxacin	1	0.01	1	48,111	4018	856	737 (97.18)
		2	0.01	1	46,074	3789	792	
	Enrofloxacin	1	0.06	2	51,832	3763	798	710 (96.24)
		2	0.06	2	51,242	3703	759	
E. coli DH5α (pAC3)	Control	1	-	-	35,815	3235	706	629 (97.42)
		2	-	-	36,641	3419	762	
	Ciprofloxacin	1	2	1	30,789	3156	714	642 (98.66)
		2	2	1	36,371	3244	731	
	Enrofloxacin	1	2	2	26,410	3112	661	596 (98.01)
		2	2	2	29,283	3260	729	
