# Supplementary material for: Unique Features of Aeromonas Plasmid pAC3 and Expression of the Plasmid-Mediated Quinolone Resistance Genes
Source: mSphere. 2017 May 24;2(3):e00203-17. doi: 10.1128/mSphere.00203-17 (PMC5444012; doi:10.1128/mSphere.00203-17)
Supplement: TABLE S4 [file sph003172292st7.pdf]

Target gene	Sequence (forward/reverse)	Reference
qnrA	5′-TTCTCACGCCAGGATTTG-3′/5′-CCATCCAGATCGGCAAA-3′	1
qnrB	5′-GGMATHGAAATTCGCCACTG-3′/5′-TTYGCBGYYCGCCAGTCG-3′	1
qnrC	5′-TGCAGACCTACGAGATGCTT-3′/5′-CGCATTCTCTCAATTCAAGG-3′	1
qnrD	5′-AAGTGCGAACTGTGGGAAA-3′/5′- CAGCCAAAGACCAATCAAAC-3′	1
qnrS	5′-GTGAGTAATCGTATGTACTTTTGC-3′/5′-AAACACCTCGACTTAAGTCT-3′	1
qnrVC1	5′-GATTGGTGCAAATCTTCGAG-3′/5′-TGGCTTAAATCACAGCCTTG-3′	1
qnrVC4	5′-ATTGGCGCAAATCTGAGTG-3′/5′-CAAATCACAACCTTGCAACC-3′	1
aac(6′)-Ib-cr	5′-TTGCGATGCTCTATGAGTGGCTA-3′/5′-CTCGAATGCCTGGCGTGTTT-3′	2
qepA	5′-CGTGTTGCTGGAGTTCTTC-3′/5′-CTGCAGGTACTGCGTCATG-3′	1
oqxA	5′-CTTGCACTTAGTTAAGCGCC-3′/5′-GAGGTTTTGATAGTGGAGGTAGG-3′	3, 4
oqxB	5′-GCGGTGCTGTCGATTTTA-3′/5′-TACCGGAACCCATCTCGAT-3′	3, 4
gyrA	5′-CGACCTTGCGAGAGAAAT-3′/5′-GTTCCATCAGCCCTTCAA-3′	5
gyrB	5′-TCCGGCGGTCTGCACGGCGT-3′/5′-TTGTCCGGGTTGTACTCGTC-3′	6
parC	5′-TCCTATCTTGATTACGCCATG-3′/5′-CATGCCATACCTACCGCGAT-3′	6

1. Flach CF, Johnning A, Nilsson I, Smalla K, Kristiansson E, Larsson DG. 2015. Isolation of novel IncA/C and IncN fluoroquinolone resistance plasmids from an antibiotic-polluted lake. J Antimicrob Chemother 70:2709-17.

2. Park CH, Robicsek A, Jacoby GA, Sahm D, Hooper DC. 2006. Prevalence in the United States of *aac(6')-Ib-cr* encoding a ciprofloxacin-modifying enzyme. Antimicrob Agents Chemother 50:3953-5.

3. Liao X, Fang L, Li L, Sun J, Li X, Chen M, Deng H, Yang Q, Li X, Liu Y. 2015. Characterization of chromosomal *qnrB* and *ampC* alleles in *Citrobacter freundii* isolates from different origins. Infect Genet Evol 35:214-20.

4. Liu BT, Wang XM, Liao XP, Sun J, Zhu HQ, Chen XY, Liu YH. 2011. Plasmid-mediated quinolone resistance determinants *oqxAB* and *aac(6')-Ib-cr* and extended-spectrum beta-lactamase gene $bla_{CTX-M-24}$ co-located on the same plasmid in one *Escherichia coli* strain from China. J Antimicrob Chemother 66:1638-9.

5. Shakir Z, Khan S, Sung K, Khare S, Khan A, Steele R, Nawaz M. 2012. Molecular characterization of fluoroquinolone-resistant *Aeromonas* spp. isolated from imported shrimp. Appl Environ Microbiol 78:8137-41.

6. Arias A, Seral C, Gude MJ, Castillo FJ. 2010. Molecular mechanisms of quinolone resistance in clinical isolates of *Aeromonas caviae* and *Aeromonas veronii* bv. *sobria*. Int Microbiol 13:135-41.
